# Modern insulins, old paradigms and pragmatism: choosing wisely when deciding how to treat type 1 diabetes

**DOI:** 10.1186/s13098-015-0033-4

**Published:** 2015-04-22

**Authors:** Beatriz D Schaan, Rafael Selbach Scheffel

**Affiliations:** Endocrine Division, Hospital de Clínicas de Porto Alegre and Medical Faculty, Universidade Federal do Rio Grande do Sul, Rua Ramiro Barcelos 2350, prédio 12, 4° andar, Porto Alegre, RS 90035-003 Brazil; Internal Medicine Division, Hospital de Clínicas de Porto Alegre, Porto Alegre, Brazil

## Abstract

There is a clinical imperative to improve metabolic control in the treatment of patients with type 1 diabetes, but in doing so, hypoglycemia should be avoided at all costs. Insulin analogues and the assumption they would better mimic the pharmacokinetic profile of endogenous insulin secretion emerged as a magic bullet in the treatment of patients with type 1 diabetes. However, although insulin analogues have pharmaceutical properties, such as pharmacodynamic stability, reproducibility of action, and a more physiological timing of action, which could possibly facilitate insulin use, the results obtained in clinical practice have not been as good as expected. Like all clinical decisions, the decision regarding which insulin would be better for the patient should be, if possible, evidence based. Here, we briefly discuss evidence for the use of insulin analogues and the different views with respect to the available evidence that lead to different interpretations and decisions regarding the use of this new technology.

## Background

Compared with the general population, at age 20 years, life expectancy in patients with type 1 diabetes is shorter by approximately 11 years for men and 13 years for women [1]. Early in life, excess mortality is explained by diabetic ketoacidosis and hypoglycemia; later, cardiovascular diseases are the main cause. However, intensive treatment is probably able to amend the chances of survival, as has been shown in the long-term follow-up of the Diabetes Control and Complications Trial (DCCT): after 27 years, mortality was significantly lower among those who had received intensive *vs.* conventional therapy [2].

The results of the DCCT drove the need to attain lower glucose levels to achieve an HbA1c of 7.0%. Nevertheless, strict glycemic control is associated with a higher incidence of hypoglycemia, an undesired and harmful side effect of treatment [3], which, by itself, can lead to fear, anxiety, poor sleep quality, loss of work productivity, impaired functioning the following day, and treatment nonadherence [4-6]. Paradoxically, nonadherence is linked to worse glycemic control, hypoglycemia and all-cause mortality [7,8]. Additionally, any form of hypoglycemia has a negative impact on quality of life, especially nocturnal and/or severe hypoglycemia [9,10].

Therefore, improved metabolic control needs to be accomplished while avoiding hypoglycemia, since diabetes management that minimizes hypoglycemia while maintaining good glycemic control would positively affect clinical outcomes, with the best result for the patient, the doctor, and the healthcare system. Great advances have been made over the last century in terms of the technologies available to treat diabetes, with glucose measurement devices, insulin pens, insulin pumps and modified insulins, among others, providing the possibility of greater metabolic control with fewer hypoglycemic episodes and better quality of life. Nevertheless, poor access to those technologies, especially among low and middle-income countries, remains the rule [11].

Here, we briefly discuss evidence for the use of insulin analogues and the different views with respect to the available evidence that lead to different interpretations and decisions regarding the use of this new technology.

## Main text

A few years after the first insulin preparations were launched on the market, long-acting insulins were developed (NPH, lente and ultralente insulins) and later animal insulins were replaced by biosynthetic human insulins. Long-acting insulins allowed patients to be treated with one or two daily injections, but because of their slow and erratic absorption, delayed clearance, and unstable basal plasma levels, hyperglycemia and hypoglycemic peaks were common with their use [12]. Genetically modified insulins (lispro, aspart, glargine and detemir) became available 50 years later [13]. Since their launch, the use of insulin analogues has steadily increased [14]. Insulin analogues more closely mimic the pharmacokinetic profile of endogenous insulin, leading to the widespread assumption that they would substantially improve the treatment of patients with type 1 diabetes. Modern insulin regimens focus on maintaining stable basal insulin levels (basal) while reducing post-prandial glucose excursions by using rapid-acting insulins (bolus) whenever necessary, in an effort to mimic the secretion pattern of endogenous insulin [15]. However, although insulin analogues have pharmaceutical properties, such as pharmacodynamic stability, reproducibility of action, and a more physiological timing of action, that may facilitate insulin use, the results obtained in randomized controlled trials (RCTs) and systematic reviews of RCTs have not been as good as originally expected.

Like all clinical decisions, the decision regarding which insulin would be better for the patient should be evidence-based. The first step in evidence-based medicine is to search for studies that answer a clinical question. Once obtained, the evidence needs to be critically examined and then, once accepted, finally, integrated with the physician’s clinical expertise and the patient’s preferences and values. Randomized controlled trials and systematic reviews of RCTs are the gold standard tools for evaluating interventions [16]. Systematic reviews that included RCTs performed in type 1 diabetic patients comparing human insulins (NPH or regular) with insulin analogues (long-acting or short-acting) failed to show any important clinical benefit considering HbA1c as the outcome [17-24]. However, in the same studies, reductions in severe and nocturnal hypoglycemia were found, especially with short-acting insulin analogues as compared to regular insulin and detemir as compared to NPH. These findings should be viewed with caution since none of the studies was blinded and most of them are of low-moderate quality and were funded by the pharmaceutical industry. Furthermore, throughout the studies, hypoglycemia was treated as a secondary endpoint and different definitions were applied.

It is also important to note that most of the patients selected to participate in these trials were not those who would most benefit from insulin analogues. Ideally, the candidates for such trials should be patients with type 1 diabetes, who have tried all strategies to attain good metabolic control with acceptable rates of hypoglycemia, including frequent glucose monitoring, adjusting insulin dosages according to changes in meals and exercise, and who have been followed closely by a healthcare team. Moreover, patients with a high risk of hypoglycemia, especially severe and nocturnal hypoglycemia, would be more likely to benefit [7]. Unfortunately, such patients are often excluded from RCTs. To date, only one RCT has been conducted in this subgroup of patients at high risk for hypoglycemia, with hypoglycemia as the primary endpoint. It found that patients randomized to the treatment with insulin analogues (detemir and aspart) experienced less hypoglycemia than those randomized to treatment with human insulins (NPH and regular) [25].

These data can be interpreted in several ways depending on the perspective of the observer, who may take a: passionate, biased, strict or pragmatic view – we include ourselves in the latter group. Passionate eyes have faith in what they believe irrespective of any diversion, scientific or otherwise, from the first impression. Biased eyes are those influenced by other interests. Clearly, a commonplace example of this view is the influence the pharmaceutical industry has on professional judgment in many medical decisions [26,27]. In general, these two patterns of interpretation would tend to view insulin analogues as being superior to human insulins. A strict interpretation is one in which only evidence, in the very strict sense of the word, is taken into account. No concessions are made, and the evidence is applicable only to those patients that are similar to those included in the RCTs published. Lastly, but no less importantly, there is the pragmatic view, which suggests that because the published RCTs do not answer all our clinical questions, the physician needs to apply his/her experience. While insulin analogues may or may not be better than human insulins, clinical practice (the specialist opinion) clearly shows they provide great benefits, especially and maybe only for the small percentage of patients that experience frequent, severe or nocturnal hypoglycemia. Such patients might be prevented from achieving greater metabolic control because their glucose levels cannot be brought down due to episodes of hypoglycemia that preclude them from achieving optimal metabolic control. Under these exceptional circumstances, common sense might be applied when considering the potential risks and benefits of interventions. The example is the classic one of the need for a control group to test the efficacy of using a parachute after jumping from an airplane [28]. If no RCT has been carried out among patients in circumstances sufficiently similar to those of our patient/s, we must look for the next best evidence and work from there. This should be called “clinical-significance based medicine”.

Another challenge is to contextualize new technologies, their high cost, evidence based medicine, the needs of patients and the ability of the healthcare system to pay for them democratically. The UK National Institute for Health and Care Excellence (NICE) and the Pharmaceutical Management Agency (PHARMAC) in New Zealand have recommended human insulins as first line therapy, but insulin analogues can be prescribed under specific circumstances, as an option for those patients with type 1 diabetes whose lifestyle is significantly restricted by recurrent symptomatic hypoglycemic episodes [29,30]. In Brazil, only a few states provide insulin analogues for patients with type 1 diabetes under multiple and varying criteria [31]. This system generates inequalities, as patients with the same clinical condition may or may not receive the benefit from the healthcare system depending on the state they live in. This is not in accordance with the principles of the Brazilian Public Healthcare System. Curiously, although insulin analogues are twice the price of human insulins in other countries, in Brazil glargine and detemir are 377% and 536% more expensive than human insulins, respectively [14,32]. Interestingly, two Brazilian states, Rio Grande do Sul and Minas Gerais, have protocols to indicate the use of the analogue glargine. In these two states the total population with type 1 diabetes is estimated to be 241000 subjects and only 5572 (2.31%) of whom meet the inclusion criteria for glargine use and are receiving this analogue.

Furthermore, there is a need to recognize that, by themselves, new technologies are certainly insufficient to ensure the goals related to diabetes are achieved. Education and support from healthcare professionals, patients and families are all-important, and may well be cheaper, and perhaps more effective in achieving better long-term outcomes [33,34]. Suboptimal control may be associated with social difficulties, inadequate food intake and mainly non-adherence to insulin plans [13,35]. There is an urgent need to invest in actions to improve these issues.

## Conclusion

In conclusion, we suggest adopting a pragmatic view of the evidence when considering the best treatment for patients with type 1 diabetes in order to achieve the lowest HbA1c with the lowest rate of hypoglycemia. We also suggest use, not only the concepts of evidence-based medicine but also of “clinical-significance based medicine”. In our opinion, clearly, while treatment with insulin analogues should not be interpreted as a magic bullet (passionate view), there is no need to wait for the perfect study to appear in order to decide to prescribe these drugs to a subgroup of patients. When recommending their use, the capacity of the healthcare system to provide it to all subjects with the same disease, without inequalities, must be considered. Protocols for wisely choosing those patients who would most probably benefit from insulin analogues are available and should guide Brazilian physicians in their prescription [36]. The equation is not simple, and all actors involved should work to achieve a proper balance.
